# Ccr4–Not complex reduces transcription efficiency in heterochromatin

**DOI:** 10.1093/nar/gkac403

**Published:** 2022-05-30

**Authors:** Pablo Monteagudo-Mesas, Cornelia Brönner, Parastou Kohvaei, Haris Amedi, Stefan Canzar, Mario Halic

**Affiliations:** Gene Center, Ludwig-Maximilians-Universität München, Munich, Germany; Gene Center, Ludwig-Maximilians-Universität München, Munich, Germany; Gene Center, Ludwig-Maximilians-Universität München, Munich, Germany; Gene Center, Ludwig-Maximilians-Universität München, Munich, Germany; Gene Center, Ludwig-Maximilians-Universität München, Munich, Germany; Department of Structural Biology, St. Jude Children's Research Hospital, 263 Danny Thomas Place, Memphis, TN 38105, USA

## Abstract

Heterochromatic silencing is thought to occur through a combination of transcriptional silencing and RNA degradation, but the relative contribution of each pathway is not known. In this study, we analyzed RNA Polymerase II (RNA Pol II) occupancy and levels of nascent and steady-state RNA in different mutants of *Schizosaccharomyces pombe*, in order to quantify the contribution of each pathway to heterochromatic silencing. We found that transcriptional silencing consists of two components, reduced RNA Pol II accessibility and, unexpectedly, reduced transcriptional efficiency. Heterochromatic loci showed lower transcriptional output compared to euchromatic loci, even when comparable amounts of RNA Pol II were present in both types of regions. We determined that the Ccr4–Not complex and H3K9 methylation are required for reduced transcriptional efficiency in heterochromatin and that a subset of heterochromatic RNA is degraded more rapidly than euchromatic RNA. Finally, we quantified the contribution of different chromatin modifiers, RNAi and RNA degradation to each silencing pathway. Our data show that several pathways contribute to heterochromatic silencing in a locus-specific manner and reveal transcriptional efficiency as a new mechanism of silencing.

## INTRODUCTION

Heterochromatin is essential to maintain genome stability and transcriptional regulation. Defects in heterochromatin formation lead to aberrant centromere and telomere function, aneuploidy and cancer. In fission yeast, constitutive heterochromatin is established at centromeres, subtelomeres and the silent mating type (*mat*) locus (1). At centromeric repeats, RNAi is essential for heterochromatin formation as Argonaute, guided by small RNAs, recruits the H3K9 methyltransferase complex CLRC to chromatin (2–8). This leads to deposition of the repressive H3K9 methylation (H3K9me) mark by Clr4, recruitment of HP1 proteins Chp2 and Swi6, and heterochromatin formation (1,3,5).

Current data suggest that heterochromatic silencing occurs through a combination of transcriptional silencing and RNA degradation. At the level of transcriptional silencing, HP1 proteins bind H3K9me nucleosomes and recruit downstream-acting complexes (9–11). HP1 protein Chp2 recruits the complex SHREC to deacetylate chromatin and to remodel nucleosomes in heterochromatin, which is required for silencing (10,12,13). These activities were suggested to reduce RNA Pol II access (14,15).

Heterochromatin is also thought to promote recruitment of the RNA degradation machinery to degrade nascent transcripts (4,16–22). In fission yeast, RNA Pol II-transcribed heterochromatic transcripts are polyadenylated (pA) products and undergo degradation by the RNAi pathway, by the Ccr4–Not complex and by the exosome (16). The first step of mRNA degradation is generally shortening of the 3′ pA tail by the Ccr4–Not complex and Pan nucleases (23). This induces removal of the 5′ cap which enables 5′-3′ exonucleases Xrn1/2 (Exo2 and Dhp1 in *S. pombe*) and 3′-5′ degradation by the exosome (Rrp6) (24).

How transcriptional silencing and RNA degradation pathways collaborate and their relative contributions to silencing are not known. In this study, we quantified the contribution of these pathways to heterochromatic silencing by analyzing RNA Pol II occupancy, nascent RNA and steady-state RNA in different fission yeast mutants. We also defined the heterochromatic factors that contribute to transcriptional silencing and/or RNA degradation. We found that transcriptional silencing occurs through reduced RNA Pol II accessibility, as previously proposed, but, unexpectedly, also through reduced transcriptional efficiency, a mechanism not previously implicated in silencing. Our data revealed that RNA Pol II transcriptional output is lower at heterochromatic loci compared to euchromatic loci relative to levels of RNA Pol II occupancy. We determined that the Ccr4–Not complex and H3K9 methylation are essential for the reduced transcriptional efficiency at heterochromatin and quantified the contributions of heterochromatin factors to the reductions in RNA Pol II occupancy, transcriptional efficiency and RNA stability.

## MATERIALS AND METHODS

### Strain construction

All *S. pombe* strains used in this study are listed in Supplemental Table S1. Strains were generated like described in (16). The strains were constructed by electroporation (Biorad MicroPulser program ShS) with a PCR-based gene targeting product leading to deletion of specific genes. For genomic integration, a PCR with long overhang primers according to Bähler *et al.* (25) was performed and the product transformed. Positive transformants were selected on YES plates containing 100–200 μg/ml antibiotics and were confirmed by PCR and sequencing.

### total RNA isolation

Total RNA was isolated of a 2 ml yeast culture with OD_600_ of 1.0 applying the hot phenol method. The pellet was resuspended in 500 μl lysis buffer (300 mM NaOAc pH 5.2, 10 mM EDTA, 1% SDS) and 500 μl phenol-chloroform-isoamylalcohol (25:24:1, Roth) and incubated at 65°C for 10 min with constant mixing. The organic and aqueous fractions were separated by centrifugation at 20 000 × g for 10 min. Nucleic acids in the aqueous fraction were precipitated with ethanol and then treated with DNAse I (Thermo Scientific) for 1 h at 37°C. DNAse was removed by a second phenol-chloroform-isoamylalcohol extraction and ethanol precipitation.

### poly(A) RNA sequencing

The poly(A) RNA library was obtained using the NEBNext Ultra II Directional RNA Library Prep Kit for Illumina (NEB) including the NEBNext Poly(A) mRNA Magnetic Isolation Module. Libraries were sequenced on Illumina HiSeq platform.

### Chromatin immunoprecipitation sequencing (ChIP-seq)

50 ml yeast cultures with an OD_600_ of 1.2 were cross-linked with 1% formaldehyde (Roth) for 15 min at room temperature. The reaction was quenched with 125 mM glycine for 5 min. The frozen pellet was resuspended in 500 μl lysis buffer (250 mM KCl, 1× Triton-X, 0.1% SDS, 0.1% Na-desoxycholate, 50 mM HEPES pH 7.5, 2 mM EDTA, 2 mM EGTA, 5 mM MgCl_2_, 0.1% Nonidet P-40, 20% glycerol) with 1 mM PMSF and Complete EDTA free Protease Inhibitor Cocktail (Roche). Lysis was performed with 0.25–0.5 mm glass beads (Roth) and the BioSpec FastPrep-24 bead beater (MP-Biomedicals), 8 cycles at 6.5 m/s for 30s and 3 min on ice. DNA was sheared by sonication (Bioruptor, Diagenode) 35 times for 30 s with a 30 s break. Cell debris was removed by centrifugation at 13 000 × g for 15 min. The crude lysate was normalized based on the RNA and Protein concentration (Nanodrop, Thermo Scientific) and incubated with 1.2 μg immobilized (Dynabeads Protein A, Thermo Scientific) antibody against Anti-RNA polymerase II CTD repeat YSPTSPS (phospho S2) antibody - ChIP Grade (ab5095, abcam) for at least 2 h at 4°C. The resin with immunoprecipitates was washed five times with each 1 ml of lysis buffer and eluted with 150 μl of elution buffer (50 mM Tris–HCl pH 8.0, 10 mM EDTA, 1% SDS) at 65°C for 15 min. Cross-linking was reversed at 95°C for 15 min and subsequent RNase A (Thermo Scientific) digest for 30 min followed by Proteinase K (Roche) digest for at least 2 h at 37°C. DNA was recovered by phenol–chloroform–isoamylalcohol (25:24:1, Roth) extraction with subsequent ethanol precipitation. For sequencing, a ChIP-seq library was made using the NEBNext Ultra II DNA Library Prep Kit for Illumina kit (NEB). Libraries were sequenced on Illumina HiSeq platform.

### Pol II bound nascent RNA sequencing (RIP-seq)

RNA IP was performed like ChIP but without RNase A digest, using Anti-RNA polymerase II CTD repeat YSPTSPS (phospho S2) antibody—ChIP Grade (ab5095, abcam). After phenol-chloroform-isoamylalcohol extraction, DNA was digested with DNAse I (Thermo Scientific) for 2 h at 37°C. RNA was recovered with a second phenol-chloroform-isoamylalcohol purification and ethanol precipitation. Sequencing libraries were produced using the NEBNext Ultra II Directional RNA Library Prep Kit for Illumina (NEB). Libraries were sequenced on Illumina HiSeq platform.

### Analysis of sequencing data

Sequencing reads obtained in the poly(A) RNA sequencing (pA RNA), Pol II ChIP, nascent RNA sequencing (Pol II RIP) and total RNA sequencing experiments were mapped to the *S. pombe* reference genome (PomBase, release 2018) using splice-aware alignment tool STAR version 2.7.3a (26). Alignment of RIP-seq and RNA-seq data was performed with STAR default parameters. Unspliced alignments of ChIP-seq data was enforced through parameters ‘–alignIntronMax 1’ and ‘–alignEndsType EndToEnd’. Reads mapping to ribosomal RNA have been removed from further analysis.

Genomic read counts were obtained using a custom script that extended basic htseq-count (27) functionality with ‘–mode intersection-strict’ option. Additionally, for RNA assays pA RNA, Pol II RIP and total RNA, we used the ‘–stranded yes’ option to identify the strand the read originated from. Only reads that mapped uniquely and reads that mapped to less than 16 locations within heterochromatic regions were counted. We chose 16 as multi-mapping threshold for heterochromatic genes to eliminate low-complexity reads without discarding reads originating from heterochromatic regions (dg/dh have 12–13 copies). We did not try to resolve the origin of multi-mapping reads, but instead counted all reads that mapped to one representative copy of dg/dh. We normalized gene counts by gene length and sequencing depth using Transcripts Per Million (TPM). Finally, average TPM values were computed for protein-coding and heterochromatic genes across biological replicates with a Spearman correlation coefficient of log transformed gene expression values of at least 0.8.

For the analysis in Supplemental Figure S1B, we obtained relative intronic read counts by first counting the number of reads overlaping each intronic region, normalizing this count by the length of the intron, and finally dividing this normalized count by the total number of reads mapping to the corresponding gene, normalized again by the gene length.

**Figure 1.**
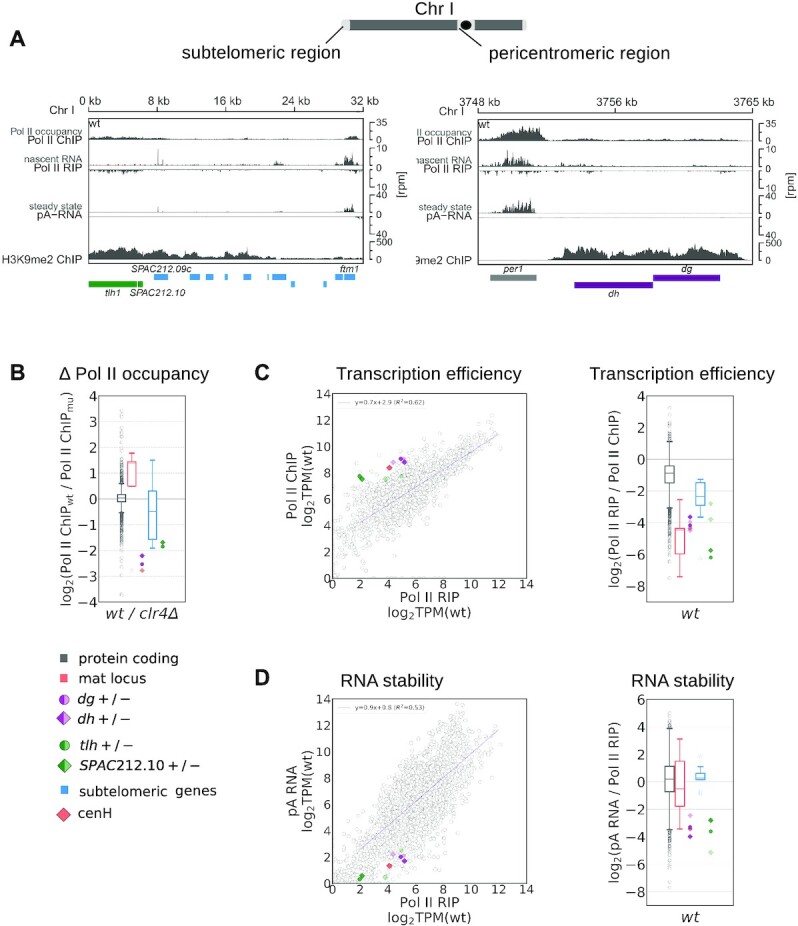
Heterochromatic repeats have reduced RNA Pol II occupancy, Transcription Efficiency and RNA stability. (**A**) Analysis of the next-generation sequencing data showing occupancy of S2P-RNA Pol II (ChIP-seq), nascent RNA (S2P-Pol II RIP-seq), steady-state RNA levels (pA RNA-seq) and H3K9me2 levels (ChIP-seq) at subtelomeric and pericentromeric regions in *S. pombe* wild-type cells. Gene locations are indicated as boxes below the coverage and color-coded: gray, protein-coding genes; purple, centromeric *dg, dh*; green, subtelomeric loci *tlh* and *SPAC212.10*; blue, other subtelomeric genes. (**B**) Box plot showing RNA Pol II occupancy (S2P-Pol II ChIP-seq) in wild-type cells relative to *clr4Δ* cells for protein-coding genes (gray), mat locus (orange), and subtelomeric genes (blue). The sequencing data for *clr4Δ* are shown in Figure S4B. For protein-coding genes, individual transcript are shown as circles; bottom and top of the box correspond to lower and upper quartiles of the data, bar is the median and whiskers are median ±1.5 times interquartile range. Colored symbols on the right show centromeric *dg* and *dh* (dark purple), subtelomeric *tlh and SPAC212.10* (dark green), and cenH (orange). Solid/transparent color show + and – strand respectively. Each data point is the average of at least two independent samples. (**C**) Transcription efficiency in wild-type cells. Left, S2P-Pol II ChIP-seq (Pol II occupancy) data plotted over S2P-Pol II RIP-seq data (nascent RNA). TPM, transcripts per million. Gray circles are individual protein-coding genes; regression line is also shown in purple. Also plotted are centromeric *dg* and *dh* (dark purple for + strand, bright purple for - strand) and *tlh* and *SPAC212.10* (dark green for + strand, bright green for – strand) and cenH (orange). Each data point is the average of at least two independent samples. Right, box plot showing transcription efficiency distributions by gene categories, data are plotted and color-coded as in panel B. **(D)** RNA stability in wild-type cells. Left, pA RNA-seq (steady-state RNA) data plotted over S2P-Pol II RIP seq data (nascent RNA). TPM, transcripts per million. Data are plotted as defined for panel (C). Right, box plot showing RNA stability (pA RNA/Pol II RIP) distribution by gene categories, data are plotted and color-coded as in panel (B).

Read coverages (as shown for example in Figure 1A) were obtained using a custom python script that uses STAR read alignments as input and returns corresponding coverages in wiggle format. The script generates a coverage profile ***x_i_***, by counting the number of read alignments *x* overlaping each genomic location *i*. Multi-mapping reads in this case fractionally contribute 1/NH to corresponding locations in the coverage profile, where NH is the number locations the read maps to. For visualization we used a custom R script that normalizes each profile by sequencing depth, making them comparable across datasets. Here, sequencing depth is computed as milions of reads that map to protein-coding genes or heterochromatic regions.

### t-distributed stochastic neighbor embedding (t-SNE)

For each mutant, we created a high-dimensional vector containing Pol II occupancy and transcription efficiency log transformed TPM values of all heterochromatic genes. In order to visualize this high-dimensional data and their relationships, we used scikit-learn's (28) implementation of t-SNE with ‘perplexity = 5’ parameter, to reduce the data to two dimensions as shown in Figure 5A.

### IP-Input subtraction in ChIP-seq data

For normalization of ChIP-seq data based on Input data, we used the three core centromeric regions to define the background component of the IP data. These are the longest regions with very low transcription and we thus scale Input data to match the IP data along these regions (29). More specifically, for each of the three regions we computed for each ChIP-seq sample a coverage profile *x*_*i*_ and a coverage profile *y*_*i*_ for the corresponding IP-Input sample, and computed a scaling factor λ using the following equation:

$$\begin{equation*}\mathop \sum \limits_{i\ = \ 1}^n {x_i} - \lambda \ \mathop \sum \limits_{i\ = \ 1}^n {y_i} = \ 0,\end{equation*}$$

where n is the gene length. We take the median of the three λ values as our final scaling factor. In Supplemental Figure S1A we show that this normalization notably reduces the read coverage within these regions in wild-type cells.

### Transcription Efficiency, RNA Stability and Pol II Occupancy

For each mutant, let θ_ChIP_, θ_RIP_ and θ_pA-RNA_ denote the average TPM value of a given gene obtained from Pol II ChIP-seq, Pol II RIP-seq and pA RNA-seq experiments, respectively, as described above. We compute transcription efficiency as the amount of newly synthesised RNA (Pol II RIP) relative to the level of Pol II Occupancy (Pol II ChIP):

$$\begin{equation*}Transcription\ Efficiency\ = \frac{{{\theta _{RIP}}}}{{{\theta _{ChIP}}}}\ \end{equation*}$$

RNA levels being the result of RNA synthesis and degradation, we define RNA stability as the ratio of steady state RNA levels (pA RNA-seq) over the amount of newly synthesised RNA (Pol II RIP):

$$\begin{equation*}RNA\ Stability\ = \frac{{{\theta _{pARNA}}}}{{{\theta _{RIP}}}}\ \end{equation*}$$

Changes in Pol II occupancy were quantified by the log fold change of Pol II occupancy levels (Pol II ChIP) in each mutant (*mu*) relative to wild-type (*wt*) cells:

$$\begin{equation*}\Delta Pol\ II\ occupancy\ = lo{g_2}\ \left( {\frac{{\theta _{ChIP}^{mu}}}{{\theta _{ChIP}^{wt}}}} \right)\end{equation*}$$

As described above, we compute ratios of averaged TPM values across replicates, similar to (30), to obtain quantitative measures of transcription efficiency, RNA stability, and changes in Pol II occupancy. Since different experiments are unpaired, we visualize variability in these ratios by combining all possible pairs of replicates between the two assays involved in each of the three quantities and provide the standard error of the mean (SEM) in Supplemental Figure S2.

### Calculation of pathways' contributions to silencing

Since the transcriptional output is proportional to Pol II occupancy, transcriptional efficiency, and RNA stability, we quantify the RNA output by:

$$\begin{eqnarray*} && RNA\ output\ = \left[ {Pol\ II\ occupancy} \right]\ \cdot \nonumber\\ && \quad \left[ {Trancription\ Efficiency} \right] \cdot \left[ {RNA\ Stability} \right]\end{eqnarray*}$$

$$\begin{equation*}RNA\ output\ = {\theta _{ChIP}}\ \cdot \left( {\frac{{{\theta _{RIP}}}}{{{\theta _{ChIP}}}}} \right) \cdot \left( {\frac{{{\theta _{pARNA}}}}{{{\theta _{RIP}}}}} \right)\end{equation*}$$

## RESULTS

### Transcriptional silencing and degradation of heterochromatic RNA

To dissect the contributions of transcriptional silencing and RNA degradation to heterochromatic silencing, we analyzed RNA Pol II occupancy, nascent RNA, and steady-state RNA at heterochromatic and euchromatic regions in fission yeast (Figure 1A shows data for specific genomic regions from wild-type cells). For Pol II occupancy, we performed ChIP-seq analyses of serine 2 phosphorylated (S2P)-RNA Pol II-bound DNA. To correct for noise, we subtracted scaled input data from all RNA Pol II ChIP-seq datasets. For normalization, the background component was defined based on the centromeric central core (Cenp-A containing chromatin), a region with low occupancy in RNA Pol II ChIP-seq; we thus subtracted input so that this region would show near zero signal (Supplemental Figure S1A, see also Methods). For nascent RNA, we performed RNA-seq of S2P-RNA Pol II-bound RNA (Pol II RIP) and assessed the quality of nascent RNA by examining retention of intronic sequences, which are strongly enriched in nascent RNA (Supplemental Figure S1B, C). For steady-state RNA, we sequenced polyadenylated (pA) RNA and total RNA; both datasets showed comparable levels of heterochromatic transcripts, indicating that these are mostly polyadenylated (Supplemental Figure S1D and E).

To determine how heterochromatin changes RNA Pol II accessibility, we compared RNA Pol II occupancy in wild-type cells and in *clr4Δ* cells, which lack H3K9 methyltransferase Clr4 and thus do not have H3K9me or heterochromatin (5) (Figure 1B). Error bars for all the data are shown in Supplemental Figure S2A. We did not detect substantial differences in RNA Pol II occupancy at protein-coding genes between wild-type and *clr4Δ* cells. At heterochromatic regions, the effects varied according to the specific locus: at centromeric *dg/dh* and subtelomeric *tlh* repeats (*tlh* and SPAC212.10), RNA Pol II occupancy was ∼4-fold higher in *clr4Δ* cells compared to wild-type cells (Figure 1B and Supplemental Figure S3A, B). At other subtelomeric regions and at the mat locus, we observed smaller change in RNA Pol II occupancy in the absence of heterochromatin (Figure 1B and Supplemental Table S2).

Our data also showed that RNA Pol II complexes can be present in heterochromatic regions, but they do not always actively transcribe or produce nascent RNA. For example, in wild-type cells, heterochromatic *tlh1* and subtelomeric *ftm1* loci show similar RNA Pol II occupancy, but *ftm1* produces substantially more nascent RNA (Figure 1A). To analyze this relationship further, we plotted the nascent RNA levels over chromatin-bound RNA Pol II for individual loci in wild-type cells (Figure 1C); we also calculated the ratio between those measurements, which informs on how much nascent RNA is synthesized relative to RNA Pol II occupancy at any given locus (Figure 1C). We define this parameter as transcription efficiency. Error bars for all the data are shown in Supplemental Figure S2B.

We found a linear relationship between RNA Pol II occupancy and nascent RNA when examining ∼5000 euchromatic, protein-coding loci in wild-type cells, indicating that transcription efficiency was constant across the examined euchromatic loci. In contrast, heterochromatic loci showed lower transcriptional efficiency (8-fold on average) compared to protein-coding loci (Figure 1C), and this observation applied to all heterochromatic regions in fission yeast. Thus, our data indicate that, in wild-type cells, heterochromatic loci are less efficiently transcribed than protein-coding loci that have the same amount of RNA Pol II, suggesting that RNA Pol II does not productively transcribe heterochromatic regions.

Next, we calculated the ratio of steady-state RNA to nascent RNA, which informs on the stability of a specific transcript. Error bars for all the data are shown in Supplemental Figure S2C. Notably, we found that a subset of heterochromatic transcripts was less stable than the average transcript from a protein-coding loci (Figure 1D). This subset of unstable heterochromatic RNAs includes transcripts from centromeric *dg/dh* and subtelomeric *tlh* regions, which are known to be degraded by RNAi and by the Ccr4–Not complex, respectively (2,4,16). For transcripts derived from the other subtelomeric genes or from the *mat* locus, RNA stability was comparable to transcripts from protein-coding genes (Figure 1D), indicating that those loci primarily undergo transcriptional silencing, with no major role for RNA degradation in their silencing.

### Effect of heterochromatin on silencing pathways

Our data indicate that three different pathways can contribute to silencing of heterochromatin: RNA Pol II occupancy, transcriptional efficiency and RNA degradation. To further examine the impact of heterochromatin structure on those pathways, we compared the contributions from each of them at heterochromatic loci in wild-type and *clr4Δ* cells.

**Figure 2.**
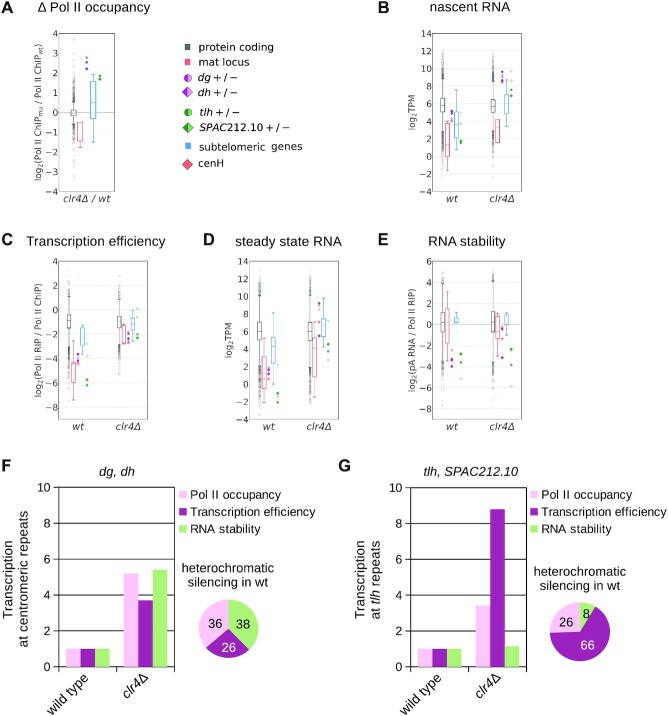
Contribution of distinct pathways to heterochromatic silencing. (**A**) RNA Pol II occupancy (Pol II ChIP-seq) in *clr4Δ* cells relative to wild-type cells for indicated gene categories; data are the same as in Figure 1B, with inverted ratios. (**B**) Nascent RNA (Pol II RIP) in wild type and *clr4Δ* cells. Data are plotted as defined for Figure 1B. (**C**) Transcription efficiency (Pol II RIP / Pol II ChIP) in wild-type and *clr4Δ* cells. Data are plotted as defined for Figure 1B. (**D**) Steady state RNA (pA RNA-seq) shown for wild-type and *clr4Δ* cells. Data are plotted as defined for Figure 1B. (**E**) RNA stability (pA RNA / Pol II RIP) in wild type and *clr4Δ* cells. Data are plotted as defined for Figure 1B. (**F, G**) Bar chart showing fold change in quantitative measures (ratios of average TPM, see Methods) of the three pathways (Pol II occupancy, transcription efficiency and RNA stability) at centromeric *dg* and *dh* (D) and at subtelomeric *tlh* (E) in *clr4Δ* cells, relative to wild type. Pie charts show relative contribution of each pathway to heterochromatic silencing at repeats in wild-type cells. Average of at least two independent samples is shown for all figures.

We found that RNA Pol II transcribes repetitive regions more efficiently in the *clr4Δ* cells compared to wild-type cells. RNA Pol II occupancy at centromeric *dg/dh* and subtelomeric *tlh* repeats was ∼four-fold higher in *clr4Δ* cells relative to wild-type cells, but the increase in nascent RNA was ∼twenty-fold (Figure 2A and B). The data also demonstrate that heterochromatin structure contributes to reduced transcriptional efficiency: in *clr4Δ* cells, all heterochromatic regions (centromeres, subtelomeres and the *mat* locus) reach transcriptional efficiency that is comparable to euchromatic regions (Figure 2C). Moreover, transcription efficiency is the single mode of heterochromatic silencing that acts in all heterochromatic regions.

We next assessed RNA stability in the absence of heterochromatin structure. We observed that subtelomeric *tlh* RNA has similarly low stability in *clr4Δ* compared to wild-type cells, indicating that multiple pathways degrade RNA from subtelomeric *tlh* repeats, independently of heterochromatin structure, in agreement with previous observations (Figure 2D and E) (16). In contrast, centromeric *dg/dh* transcripts showed increased stability in *clr4Δ* compared to wild-type cells, indicating that heterochromatin structure is required for degradation of those transcripts.

These data from *clr4Δ* cells also confirm our observations with wild-type cells: at centromeric *dg/dh*, silencing results from a combination of reduced RNA Pol II occupancy (Figure 2A), reduced transcriptional efficiency (Figure 2C) and increased RNA degradation (Figure 2E), whereas for subtelomeric *tlh* repeats, silencing occurs primarily at the transcriptional level.

We quantified the relative contribution of each pathway to heterochromatic silencing, by comparing data from wild-type and *clr4Δ* cells. In wild-type cells, RNA degradation and transcriptional silencing contribute similarly to silencing of centromeric repeats (∼38% and ∼62%, respectively) (Figure 2F) wherein transcriptional silencing can be further parsed out into RNA Pol II occupancy (∼36%) and transcriptional efficiency (∼26%). At subtelomeric *tlh* repeats, heterochromatic silencing occurs primarily by transcriptional silencing (∼92%) (Figure 2G), which is predominately mediated through reduced transcriptional efficiency (∼66%). A similar pattern is observed at the remaining subtelomeric regions and *mat* locus: at *mat* locus silencing is mostly transcriptional, with transcriptional efficiency (∼70%) being the major silencing pathway (Supplemental Figure S1F, G).

### Contribution of heterochromatin factors to distinct silencing pathways

Next, we analyzed RNA Pol II occupancy, transcriptional efficiency and RNA degradation in strains defective in the RNAi machinery (*ago1Δ*), lacking chromatin modifiers (*clr3Δ*, *mit1Δ*, *chp2Δ* and swi6Δ) or RNA degradation components (*rrp6Δ*, *exo2Δ, caf1Δ, ccr4Δ* and *mot2Δ*) (Supplemental Figure S3-S7); error bars between replicates shown in Supplemental Figure S2. Chp2 and Swi6 are HP1 family proteins; wherein Chp2 recruits the SHREC complex to chromatin. SHREC subunits, Mit1 and Clr3, are a chromatin remodeler and a histone deacetylase, respectively (10,13). Rrp6 is a component of the exosome complex; Exo2 is a 5′→3′ exonuclease. Caf1, Ccr4 and Mot2 are components of the Ccr4–Not deadenylase complex.

**Figure 3.**
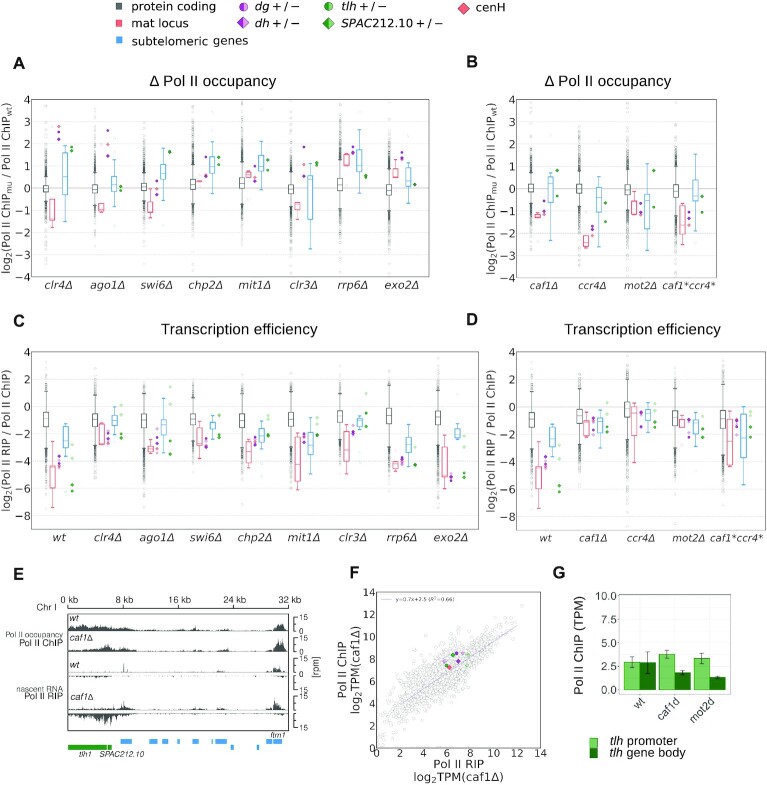
RNA Pol II occupancy and transcription efficiency in different mutants. (**A, B**) Box plots showing ratio of RNA Pol II occupancy (S2P-Pol II ChIP-seq) in mutants compared to wild type over indicated genes. Shown are mutants in factors involved in heterochromatin formation and RNA degradation (A) or Ccr4–Not complex components (B). Data are plotted as defined for Figure 1B. (**C, D**) Box plot showing transcription efficiency (Pol II RIP / Pol II ChIP) over indicated genes in wild type and mutants in factors involved in heterochromatin formation and RNA degradation (C) or in Ccr4–Not complex components (D). Data are plotted as defined for Figure 1B. (**E**) Analysis of the next-generation sequencing data showing occupancy of S2P RNA Pol II (ChIP-seq) and nascent RNA (S2P-Pol II RIP-seq) at subtelomeric regions in *S. pombe* wild-type and *caf1Δ* cells. Gene locations are indicated as boxes below the coverage and color-coded: green, subtelomeric loci *tlh* and *SPAC212.10*; blue, other subtelomeric genes. (**F**) Transcription efficiency in *caf1Δ* cells. S2P-Pol II ChIP-seq (Pol II occupancy) data plotted over S2P-Pol II RIP-seq data (nascent RNA). TPM, transcripts per million. Gray circles are individual protein-coding genes; regression line is also shown in purple. Also plotted are centromeric *dg* and *dh* (dark purple for + strand, bright purple for – strand) and *tlh* and *SPAC212.10* (dark green for + strand, bright green for - strand). Each data point is the average of at least two independent samples. (**G**) Quantification of RNA Pol II occupancy (S2P-Pol II ChIP-seq) at *tlh* promoter region and *tlh* gene body in indicated wild type and mutant strains.

Our data show that reduced RNA Pol II occupancy at centromeric repeats requires RNAi (Figure 3A and Supplemental Figure S3A, S4A–C), HP1 proteins, components of SHREC (Figure 3A and Supplemental Figure S4D, E, S5A, B) and Exo2 and Rrp6 of the RNA degradation machinery (Figure 3A and Supplemental Figure S5C-D). At subtelomeric *tlh* repeats, only chromatin modifiers are required to limit RNA Pol II occupancy (Figure 3A and Supplemental Figure S3B, S4D, E, S5A, B), whereas at the remaining subtelomeric genes and at the cenH element of the *mat* locus, RNA Pol II occupancy was increased in absence of several chromatin modifiers and *rrp6* (Figure 3A and Supplemental Figure S3C, D, S4E, S5A). Finally, the Ccr4–Not complex components showed little effect on RNA Pol II occupancy at all heterochromatic regions (Figure 3B and Supplemental Figure S6A–C).

The reduced transcriptional efficiency at heterochromatic loci depends on most chromatin modifiers (Clr4, HP1 proteins and SHREC complex), but not on RNA degradation factors Rrp6 and Exo2 (Figure 3C and Supplemental Figure S4–S6). Those chromatin modifiers seem to reduce transcriptional efficiency more than RNA Pol II occupancy (Supplemental Figure S3), an effect similar to what we had observed in *clr4Δ* cells. The strongest effect on transcriptional efficiency in heterochromatic regions was observed for components of the Ccr4–Not complex (Figure 3D and Supplemental Figure S3). The Ccr4–Not deadenylase complex could affect transcriptional efficiency indirectly, through changes in RNA levels of other factors involved in heterochromatin formation. However, analysis of nascent RNA, total RNA and pA RNA data show that RNA levels of factors involved in heterochromatin formation do not change substantially in *caf1Δ* cells compared to wild-type cells (Supplemental Figure S7E). This suggests a direct effect of Ccr4–Not on transcriptional efficiency.

Although RNA Pol II occupancy at *tlh* repeats is comparable in *caf1Δ* and wild-type cells, *caf1Δ* cells produce ∼10 fold more nascent RNA from that locus than wild-type cells (Figure 3E). Thus transcriptional efficiency is increased in all heterochromatic regions in *caf1Δ* cells, to a level comparable to protein-coding genes (Figure 3D, F). To determine if Ccr4–Not mediated reduction in transcription efficiency occurs post-heterochromatin formation, we analyzed H3K9me levels in wild-type and *caf1Δ* cells. Notably, H3K9me levels are only slightly affected at *tlh* and centromeric *dg/dh* repeats in *caf1Δ* cells (Supplemental Figure S7F), indicating that increased transcriptional efficiency does not interfere with H3K9me deposition. Thus, Caf1 reduces transcriptional efficiency at heterochromatin loci after H3K9me is deposited and heterochromatin is established; in fact, Caf1 requires H3K9me and heterochromatin for its activity. In absence of heterochromatin, transcriptional efficiency is strongly increased, as seen by our data on *clr4Δ* (Figure 2C). Moreover, in *caf1Δago1Δ* cells, which lack both H3K9me and small RNAs (16), transcriptional efficiency is not further increased (Figure S8), suggesting that Ccr4–Not is the primary cause of reduced transcriptional efficiency in heterochromatin. In sum, our data show that the Ccr4–Not complex is required to reduce transcriptional efficiency in heterochromatic regions and that it modulates RNA Pol II activity in a heterochromatin-dependent way.

To test if the deadenylase activity of the Ccr4–Not complex is required for reduction of transcriptional efficiency in heterochromatin, we introduced point mutations into the active site of the two deadenylases in the complex, Caf1 and Ccr4 (16). The mutations in the active sites led to an increase in transcriptional efficiency at heterochromatin loci that was comparable to the effect seen with gene deletions (Figure 3D), suggesting that the nuclease activity is required for reduced transcriptional efficiency. The deadenylase activity of the Ccr4–Not complex requires an accessible 3′ RNA end, which is not the case with nascent RNA, where the 3′ end is engaged with RNA Pol II. It is possible that our Pol II-RIP assay also detects chromatin-bound RNA that are targeted by the Ccr4–Not complex (16), thus contributing to reducing transcriptional efficiency. Alternatively, Ccr4–Not might bind backtracked nascent RNAs (Dutta *et al.*, 2015; Kruk *et al.*, 2011) which would have accessible 3′ ends, and this could potentially stall RNA Pol II.

In support of a direct effect on transcription, we observed changes in RNA Pol II distribution upon deletion of the Ccr4–Not complex at the *tlh* locus (Figure 3E, G and Supplemental Figure S9A). Whereas RNA Pol II occupancy at the promoter and 5′ end of the *tlh* gene in *caf1Δ* and wild-type cells is comparable (Figure 3E), we observe strong reduction of RNA Pol II occupancy in the gene body in the mutant, suggesting higher RNA Pol II processivity (Figure 3E, G). Similar changes are observed in deletions of all components of the Ccr4–Not complex (Supplemental Figure S9A), especially in *mot2Δ* cells, but not in deletions of chromatin complexes such as *swi6* or *clr3* (Supplemental Figure S9B). Notably, deletion of Ccr4–Not components did not change RNA Pol II profiles in euchromatic regions (Supplemental Figure S9C). These data indicate that the Ccr4–Not complex directly affects the RNA Pol II distribution in heterochromatic regions.

**Figure 4.**
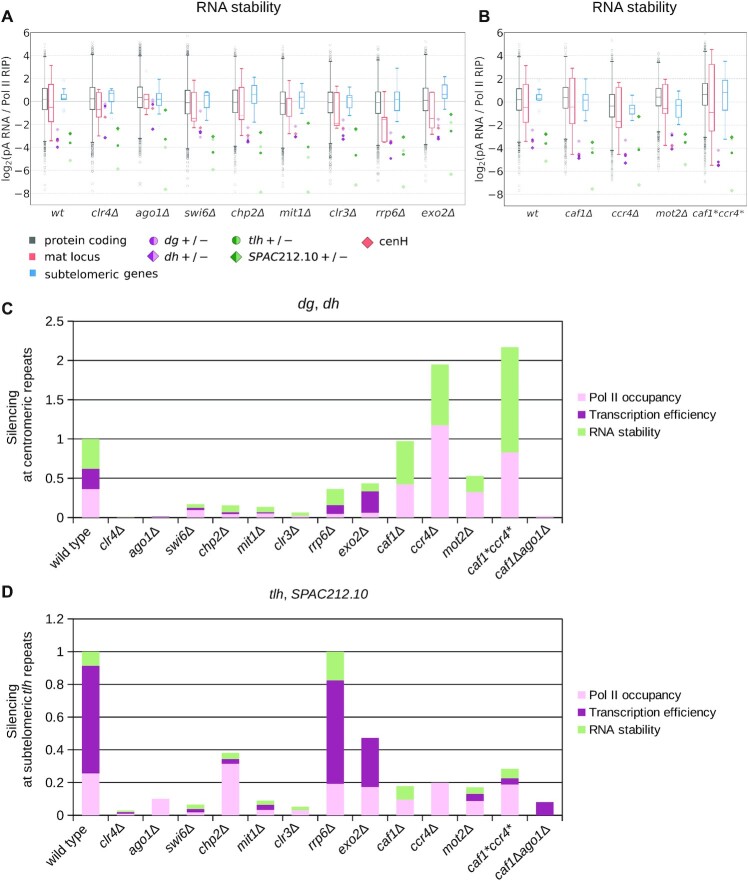
Contribution of individual factors to each pathway of heterochromatic silencing. (**A, B**) Box plot showing RNA stability (pA RNA/Pol II RIP) over indicated genes in wild type and mutants in factors involved in heterochromatin formation and RNA degradation (A) or Ccr4–Not complex components (B). Data are plotted as defined for Figure 1B. (**C, D**) Bar charts displaying contribution of each of the pathways that are still active in the mutants to silencing at centromeric *dg/dh* regions (C) and subtelomeric *tlh* regions (D). The height of each bar corresponds to the fold change in RNA output relative to wild-type. The relative contribution of each pathway was computed as fold change in quantitative measures (ratios of average TPM, see Materials and Methods) relative to *clr4Δ*.

Our data reveal that low RNA stability contributes primarily to the silencing of centromeric *dg* and *dh* transcripts (Figure 4A and Supplemental Figure S3A), with exception of *dh+* transcripts which are degraded in an RNAi- and heterochromatin-independent way. This effect requires RNAi (Ago1) and H3K9me (Clr4), but not other heterochromatic factors or individual RNA degradation factors examined (Figure 4A). Among the latter, only deletion of *exo2* showed a small increase in RNA stability at the centromeric region, suggesting that multiple RNA degradation pathways act redundantly to degrade heterochromatic transcripts (Figure 4A and Supplemental Figure S3A). In contrast, deficiency in the Ccr4–Not complex components increased degradation of heterochromatic transcripts compared to wild-type cells. This effect is consistent with increased transcriptional efficiency in this mutant, leading to higher amounts of nascent RNA (Figure 4B and Supplemental Figure S3, S9D), which would recruit the RNAi machinery to these regions, thus leading to increased degradation (16).

### Contribution of individual proteins to the heterochromatic silencing pathways

For all the mutant strains examined here, we quantified the level of silencing imposed by each of the three pathways (Pol II occupancy, transcriptional efficiency, RNA stability) at different heterochromatic loci (Figure 4C, D and Supplemental Figure S9E, F). It should be also noted that the three pathways do not operate independently in cells. The quantification of each pathway's contribution to heterochromatic silencing was calculated relative to *clr4Δ*, which was defined as a complete loss of heterochromatic silencing. We observed that Ago1 is essential for all three silencing pathways at the centromeric region; in fact, heterochromatic silencing is completely lost in strains lacking RNAi. In the strains bearing deletions of chromatin modifiers, silencing overall was strongly reduced, but each pathway was still active. In contrast, deletions of RNA degradation factors led to more limited loss of silencing, with varying effects between centromeric and subtelomeric regions.

We had previously shown that the Ccr4–Not complex degrades subtelomeric RNA redundantly with RNAi (16). Our new data show that the Ccr4–Not complex is also required for silencing at the transcriptional level, regulating transcriptional efficiency at all heterochromatic loci (Figure 4C, D and Supplemental Figure S3). Although transcriptional efficiency is increased in *caf1Δ, ccr4Δ* and *mot2Δ* cells, the overall loss of silencing at centromeric repeats is small and increased transcriptional efficiency is compensated by reduced RNA Pol II occupancy and increased RNA degradation (Figures 3B, D, 4B and Supplemental Figure S3). The loss of silencing in *caf1Δ, ccr4Δ* and *mot2Δ* cells was more pronounced at subtelomeric *tlh* repeats (compared to centromeric loci), since transcriptional efficiency is the dominant silencing pathway in those regions, and increased degradation by RNAi (16) is not sufficient to compensate for its loss (Figure 4C, D). Notably, at subtelomeric regions other than *tlh* and at the *mat* locus, the transcriptional efficiency pathway remains active in almost all mutant strains examined; the exceptions were mutants for the components of the Ccr4–Not complex, in which transcriptional efficiency is abolished but other pathways were fully functional (Supplemental Figure S9E, F). These data show that Ccr4–Not complex specifically affects transcriptional efficiency.

**Figure 5.**
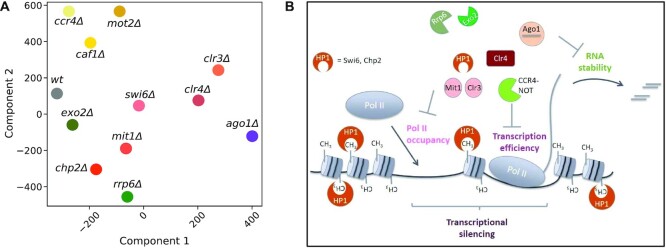
Heterochromatic silencing is a combination of reduced RNA Pol II occupancy, Transcription Efficiency and RNA stability. (**A**) The t-distributed stochastic neighbor embedding (t-SNE) plot showing two dimensional embedding of Pol II occupancy and transcription efficiency. Close proximity of mutants visualizes similarities in transcriptional silencing. (**B**) Schematic presentation of how different proteins involved in heterochromatin formation or RNA degradation contribute to heterochromatic silencing. Three pathways are important for heterochromatic silencing: transcriptional silencing (consisting of Pol II occupancy and transcription efficiency) and RNA degradation.

We projected log transformed TPM values of Pol II occupancy and transcription efficiency across all heterochromatic genes into two-dimensional space using t-distributed stochastic neighbor embedding (t-SNE). In agreement with our previous calculations (Figures 3 and 4), the t-SNE plot shows that the components of the Ccr4–Not complex *caf1, ccr4* and *mot2* co-localize, indicating their specialized role in transcriptional silencing (Figure 5A).

## DISCUSSION

Our data show that heterochromatic silencing occurs through different mechanisms. First, RNA Pol II accessibility is reduced at heterochromatic regions, which also reduces overall transcription at those loci (Figure 5B). Second, we identified transcriptional efficiency as a new mode of heterochromatic silencing; although RNA Pol II is present at heterochromatic loci, transcriptional efficiency is reduced by heterochromatin and the Ccr4–Not complex (Figure 5B). This mode of silencing operates/occurs at all heterochromatic regions in fission yeast. Third, the final component of heterochromatic silencing is RNA degradation by RNAi and several RNA degradation factors, including the Ccr4–Not complex.

Reduced RNA Pol II access was initially proposed as the major mode of heterochromatic silencing and has been observed in many organisms (31,32). In fact, we observed that RNA Pol II occupancy at centromeres strongly increased in cells bearing mutations that greatly affect H3K9me levels, such as *clr4Δ* or *ago1Δ* (33). Components of the heterochromatin pathway that act downstream of H3K9me, such as HP1 proteins, are required to reduce RNA Pol II occupancy, but to a lesser extent than Clr4. Notably, RNA degradation factors Exo2 and Rrp6 contribute to reduced RNA Pol II occupancy at the centromeric region, in agreement with our previous finding that RNA degradation is required for formation of heterochromatic domains (16).

In addition to reduced RNA Pol II occupancy, we identified reduced transcriptional efficiency as another mode of transcriptional silencing. Although RNA Pol II is present at heterochromatic regions, the RNA it produces (nascent RNA levels) is proportionally lower than in euchromatic regions. This silencing mode occurs at all heterochromatic regions in fission yeast cells and might be conserved in other organisms as well. In fact, this mode of silencing might be analogous to SIR-mediated silencing in *S. cerevisiae*, wherein transcription is initiated but elongation is blocked by the SIR complex, which maintains RNA Pol II in a stalled conformation (34).

We found that heterochromatin is essential to reduce transcriptional efficiency: in the absence of H3K9me, transcription efficiency at heterochromatic loci is increased to the level comparable to euchromatic genes. These results also show that reduced transcriptional efficiency is not encoded in the DNA sequence itself, but it is controlled by the heterochromatin. We show that reduced transcriptional efficiency is mediated by the Ccr4–Not complex, as transcriptional efficiency increased to the level of protein-coding genes in *caf1Δ* cells. Notably, H3K9me levels at subtelomeric *tlh* and centromeric *dg*/*dh* repeats were only modestly affected in *caf1Δ* cells (16,18,22), indicating that heterochromatin formation is functional in those cells. Thus, the Ccr4–Not complex regulates transcriptional efficiency post-heterochromatin formation, but H3K9me and heterochromatin are required for this regulation by the Ccr4–Not complex.

The observed reduction in transcription efficiency is likely a combination of reduced transcription by RNA Pol II and degradation of chromatin bound RNA. RNAi, Ccr4–Not and the Rix1 complex were suggested to co-transcriptionally degrade heterochromatic transcripts in fission yeast (3,7,16,35,36). In wild-type fission yeast, centromeric *dg/dh* transcripts are targeted by RNAi, whereas subtelomeric *tlh* transcripts are not. Our data show that transcriptional efficiency is reduced at both centromeric *dg/dh* transcripts and subtelomeric *tlh* transcripts, suggesting that the reduction does not occur via co-transcriptional degradation by RNAi. Moreover, in *caf1Δ* cells transcriptional efficiency in heterochromatin is comparable to protein coding genes, however, both *tlh* and centromeric *dg/dh* transcripts remain to be degraded by RNAi (16). This indicates that RNAi degradation has only a minor contribution to the observed reduction in transcriptional efficiency. Notably, our data show that mutations in the active site of the Ccr4–Not deadenylases lead to an increase in transcriptional efficiency comparable to that seen with gene deletions, suggesting that nuclease activity is required for reduced transcriptional efficiency. This also suggests that the Ccr4–Not complex might degrade chromatin-bound RNA, which might co-purify with nascent RNA.

Alternatively, the deadenylase activity of Ccr4–Not might directly regulate RNA Pol II. In support of this, we observe changes in the RNA Pol II distribution, which was reduced in *tlh* gene body upon deletion of Ccr4–Not subunits. This suggests higher processivity of elongating RNA Pol II in those mutants compared to wild-type cells. Ccr4–Not was initially described as a chromatin-associated complex involved in transcription (37), and later shown to act as a transcription elongation factor that would reactivate arrested RNA Pol II (38,39). It is plausible that, in the presence of heterochromatic marks, Ccr4–Not exhibits the opposite effect and stalls RNA Pol II, perhaps by targeting backtracked nascent RNAs that have accessible 3′ ends. Recently, Ccr4–Not was shown to be required for DNA-damage dependent ubiquitination and degradation of RNA Pol II (40), and stalling of RNA Pol II by Ccr4–Not could require RNA Pol II ubiquitination. Furthermore, the Ccr4–Not complex is recruited to RNA Pol II by the histone chaperone Spt6 (41), which was also implicated in heterochromatin formation in fission yeast (42). Altogether, these various observations support the concept that Ccr4–Not is recruited to chromatin and regulates RNA Pol II transcription.

Our data show that RNA degradation contributes to heterochromatic silencing at centromeric repeats and *tlh*, but not at other subtelomeric genes or at the *mat* locus. RNA degradation at the centromeric region is dependent on RNAi and heterochromatin, but those are not required for RNA degradation at subtelomeric *tlh* repeats. This observation is in agreement with the previous report that RNA is degraded at subtelomeric *tlh* repeats by parallel mechanisms that are heterochromatin-dependent and -independent (16).

In conclusion, we identified a new mode of heterochromatic silencing termed transcriptional efficiency. This mode of silencing depends on H3K9me and the Ccr4–Not complex and acts as a dominant silencing pathway at most heterochromatic loci in fission yeast.

## DATA AVAILABILITY

The sequencing data reported in this paper have been submitted to the NCBI Gene Expression Omnibus (GEO; http://www.ncbi.nlm.nih.gov/geo/) under the accession number GSE186273.

## SOFTWARE AVAILABILITY

The whole data processing pipeline containing Python and R code is available in the form of Jupyter notebooks at https://github.com/canzarlab/heterochr_silencing.

## Supplementary Material

gkac403_Supplemental_File
