# Supplementary material for: Ccr4–Not complex reduces transcription efficiency in heterochromatin
Source: Nucleic Acids Res. 2022 May 30;50(10):5565–76. doi: 10.1093/nar/gkac403 (PMC9177971; doi:10.1093/nar/gkac403)
Supplement: gkac403_Supplemental_File [file gkac403_supplemental_file.pdf]

Supplemental Information

Title:

Ccr4-Not complex reduces transcription efficiency in heterochromatin

Authors:

Pablo Monteagudo[1,3], Cornelia Brönner[1,3], Parastou Kohvaei[1], Haris Amedi[1], Stefan Canzar*[1], Mario Halic*[2]

Affiliations:

[1]Department of Biochemistry, Gene Center,

University of Munich LMU, 81377 Munich, Germany

[2]Department of Structural Biology, St. Jude Children's Research Hospital, 263 Danny Thomas Place, Memphis, TN, 38105, USA

[3]Equal contribution

*Corresponding authors

e-mail: mario.halic@stjude.org; canzar@genzentrum.lmu.de

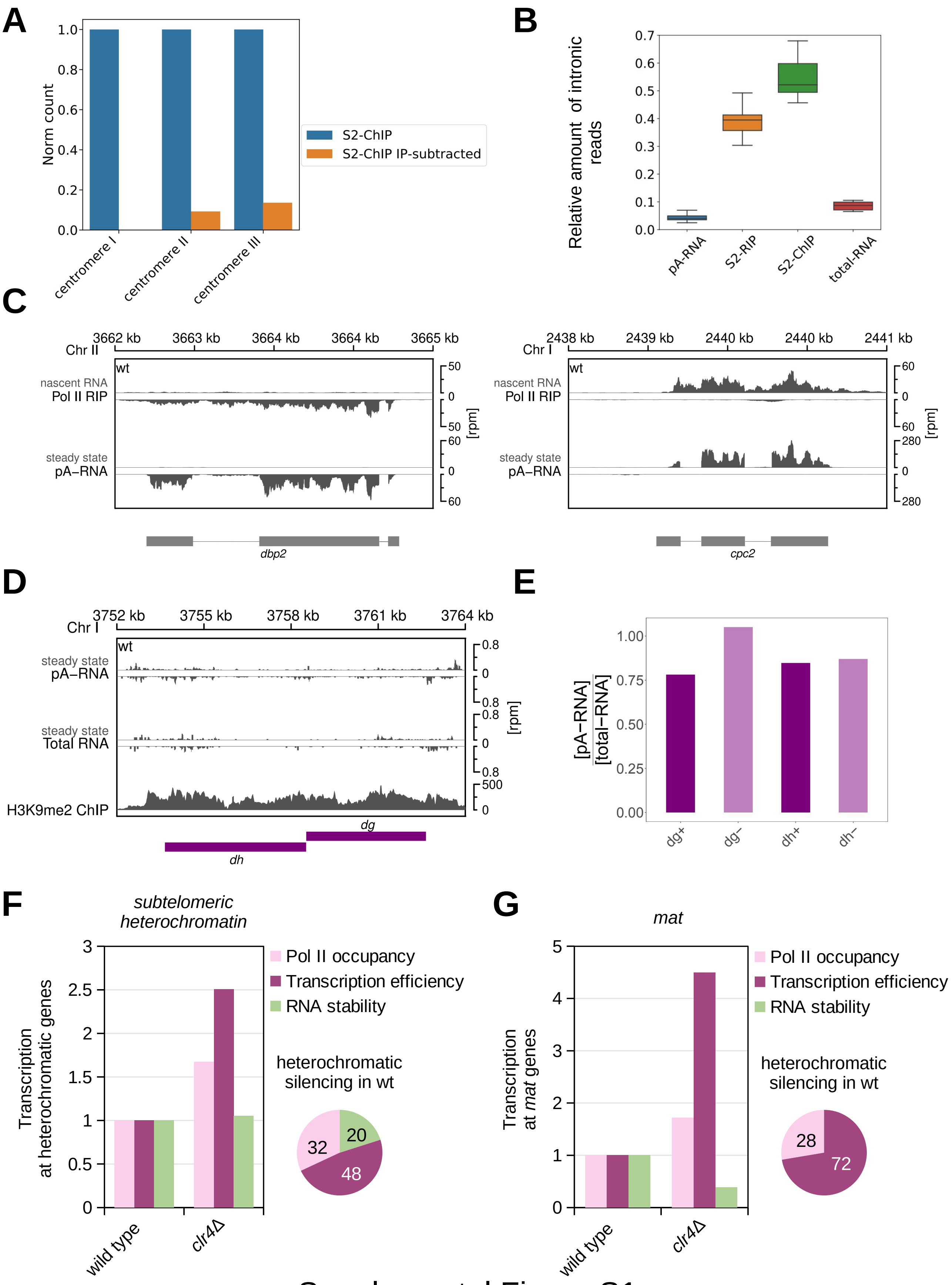

Supplemental Figure S1

Supplemental Figure S1: Silencing in wild-type cells.

(A) Analysis of S2P-Pol II ChIPseq read counts from wild type cells at centromeric central core (Cenp-A chromatin) before and after normalized input subtraction. Note that after subtraction most reads from this region, defined as noise, are removed.

(B) Quantification of intronic reads in pA RNA, total RNA, Pol II RIP and Pol II ChIP data. The data reveal high retention of intronic reads in Pol II RIP data. Bottom and top of the box correspond to lower and upper quartiles of the data, bar is the median and whiskers are median +/-1.5 times interquartile range.

(C) Analysis of the next-generation sequencing data showing comparison between nascent RNA (RIP) and total RNA (pA) sequencing. Note the presence of intronic reads in Pol II RIP data, indicating nascent RNAs.

(D) Analysis of the next-generation sequencing data showing steady state RNA levels (total RNA-seq and pA RNA seq) and H3K9me2 levels (ChIP-seq) at pericentromeric regions in *S. pombe* wild-type cells. Locations of genes are indicated as boxes below the coverage according the color code: purple = *dg, dh*.

(E) Quantification of total and pA RNA reads over *dg* and *dh* transcripts showing that *dg+/dg-* and *dh+/dh-* transcripts are similarly polyadenylated.

(**F, G**) Bar chart showing fold change in quantitative measures (ratios of average TPM, see Methods) of the three pathways (Pol II occupancy, transcription efficiency and RNA stability) at (**F**) other subtelomeric genes and (**G**) *mat* locus. Pie charts show relative contribution of each pathway to heterochromatic silencing at repeats in wild-type cells.

Average of at least two independent samples is shown for all figures.

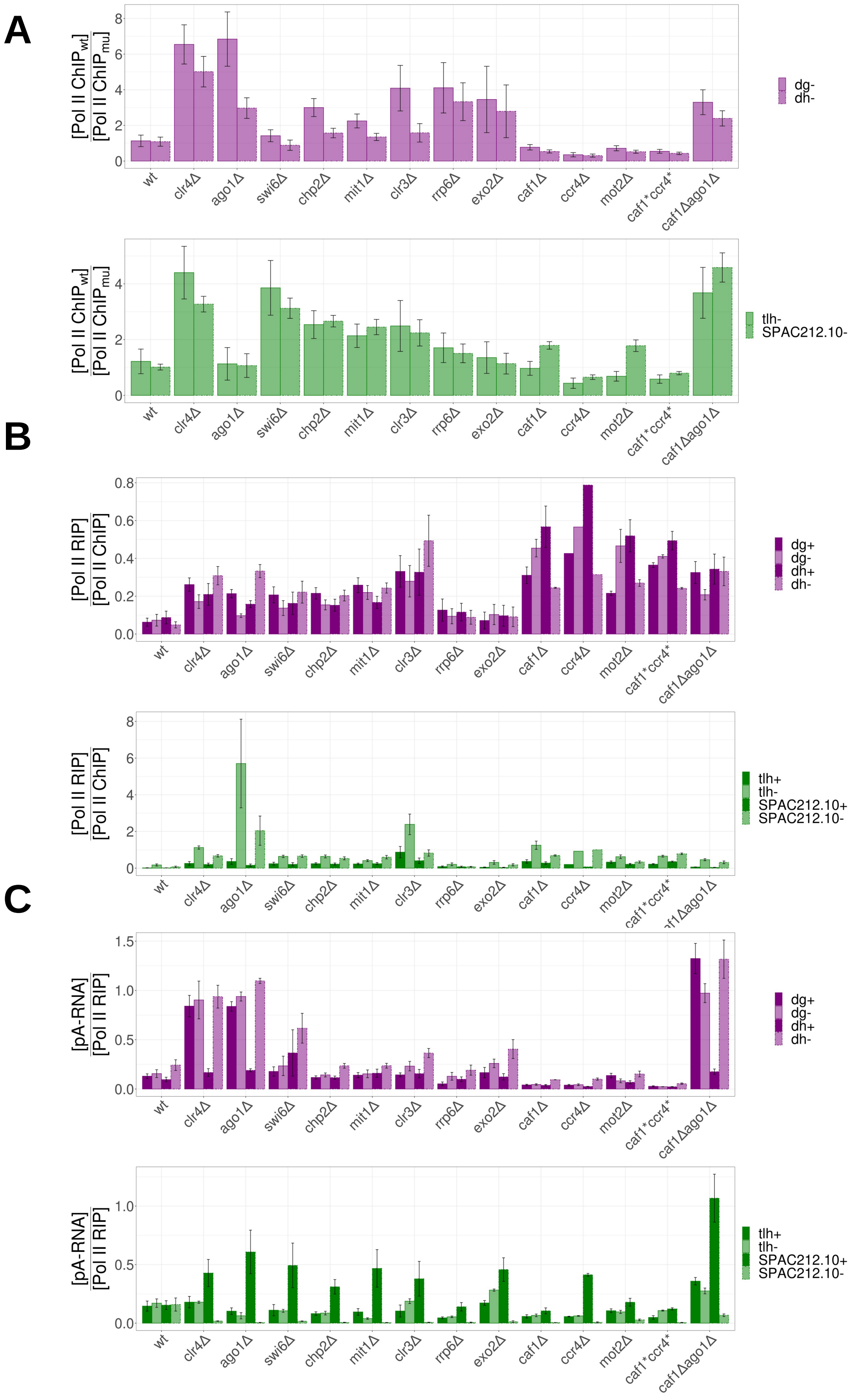

Supplemental Figure S2

Supplemental Figure S2: Confidence intervals.

Standard error between replicates for RNA Pol II occupancy, transcriptional efficiency and RNA degradation at centromeric *dg/dh* and subtelomeric *tlh* repeats.

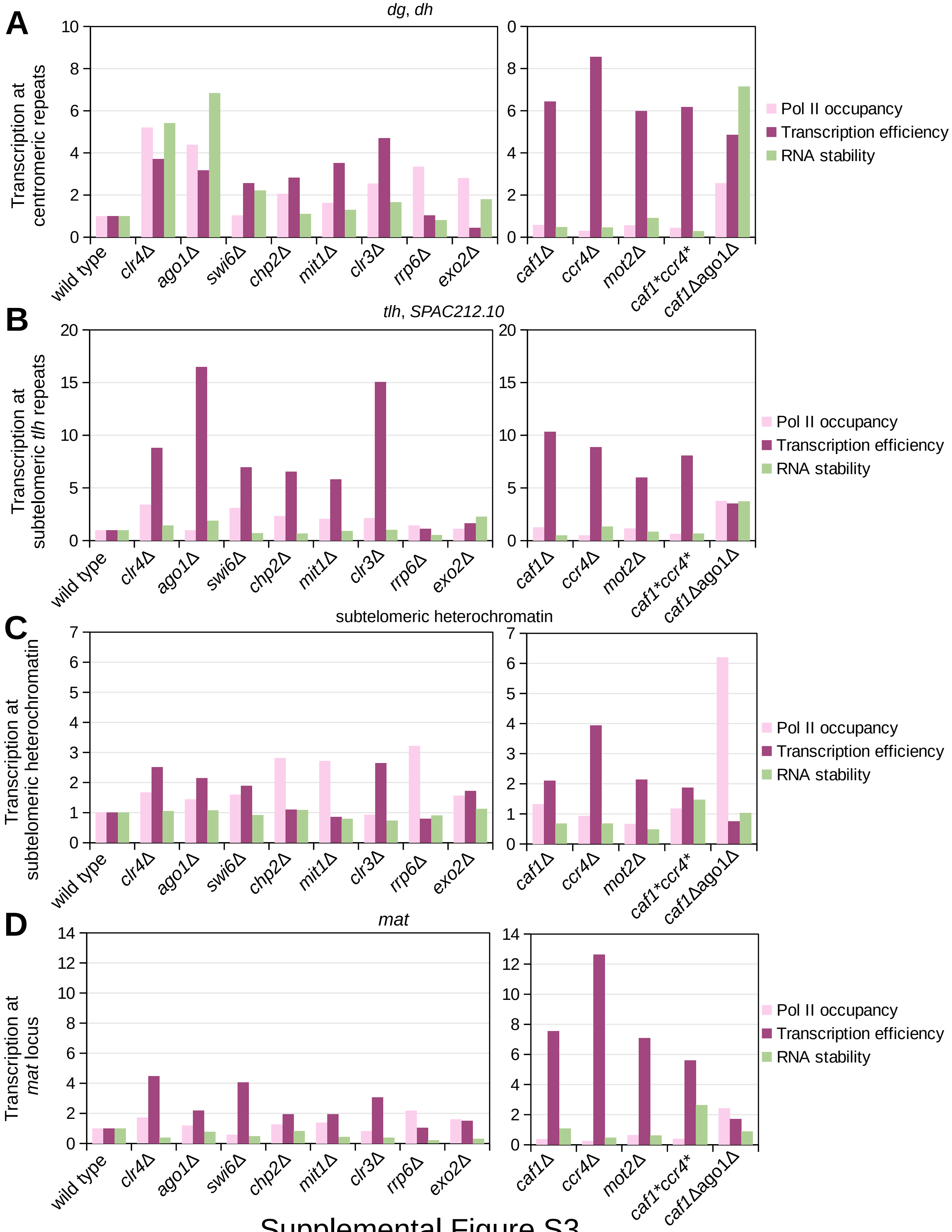

Supplemental Figure S3

Supplemental Figure S3: Silencing in mutant cells.

Bar chart showing fold change in quantitative measures (ratios of average TPM, see Methods) of the three pathways (Pol II occupancy, transcription efficiency and RNA stability) at (**A**) centromeric repeats, (**B**) subtelomeric *tlh* repeats, (**C**) other subtelomeric genes and (**D**) *mat* locus. Pie charts show relative contribution of each pathway to heterochromatic silencing at repeats in wild-type cells. Average of at least two independent samples is shown for all figures.

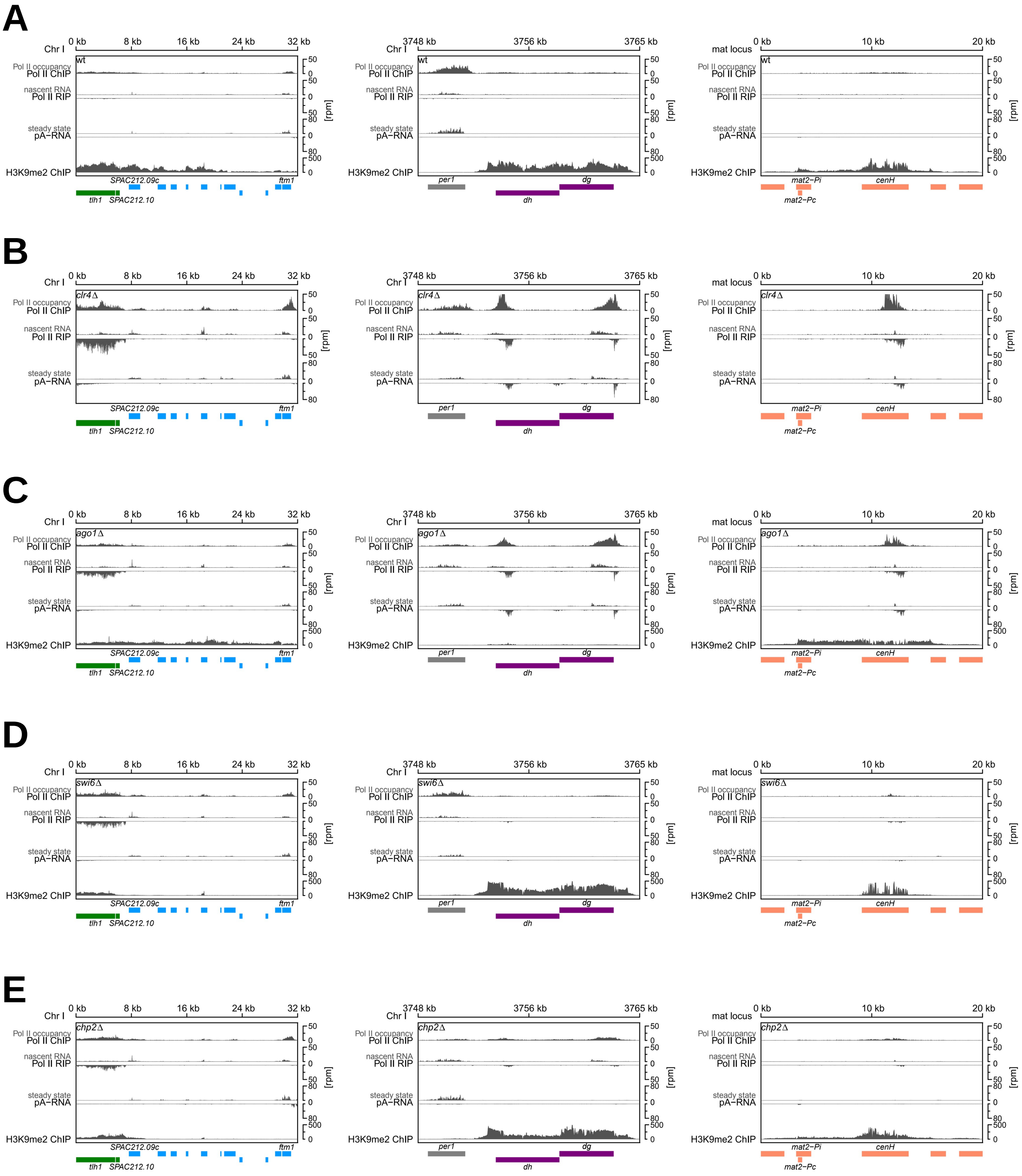

Supplemental Figure S4

Supplemental Figure S4: Next-Generation-Sequencing analysis

Next-Generation-Sequencing analysis of S2P-Pol II ChIP-seq (Pol II occupancy), S2P-Pol II RIP-seq (nascent RNA) and pA RNA-seq (steady state RNA) at subtelomeric and centromeric repeats, and at the *mat* locus. Locations of genes are indicated as boxes below the coverage according the color code: gray = protein coding; purple = *dg, dh*; green = *tlh, SPAC212.10*, blue = other heterochromatic genes, orange = *mat* locus.

(**A**) wild type (**B**) *clr4Δ* (**C**) *ago1Δ* (**D**) *swi6Δ* (**E**) *chp2Δ*

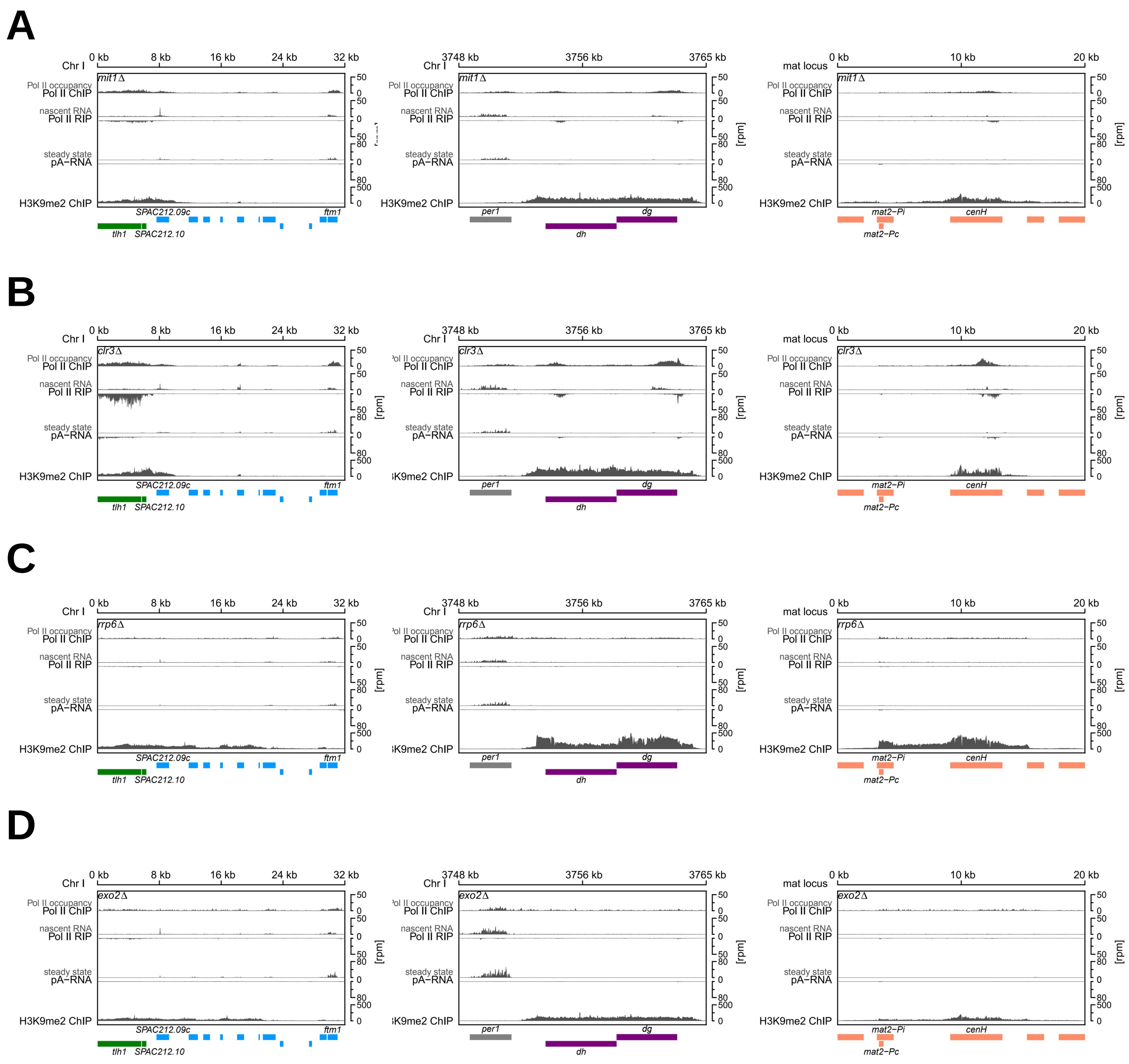

Supplemental Figure S5

Supplemental Figure S5: Next-Generation-Sequencing analysis

Next-Generation-Sequencing analysis of S2P-Pol II ChIP-seq (Pol II occupancy), S2P-Pol II RIP-seq (nascent RNA) and pA RNA-seq (steady state RNA) at subtelomeric and centromeric repeats, and at the *mat* locus. Locations of genes are indicated as boxes below the coverage according the color code: gray = protein coding; purple = *dg, dh*; green = *tlh, SPAC212.10*, blue = other heterochromatic genes; orange = *mat* locus.

(**A**) *mit1Δ* (**B**) *clr3Δ* (**C**) *rrp6Δ* (**D**) *exo2Δ*

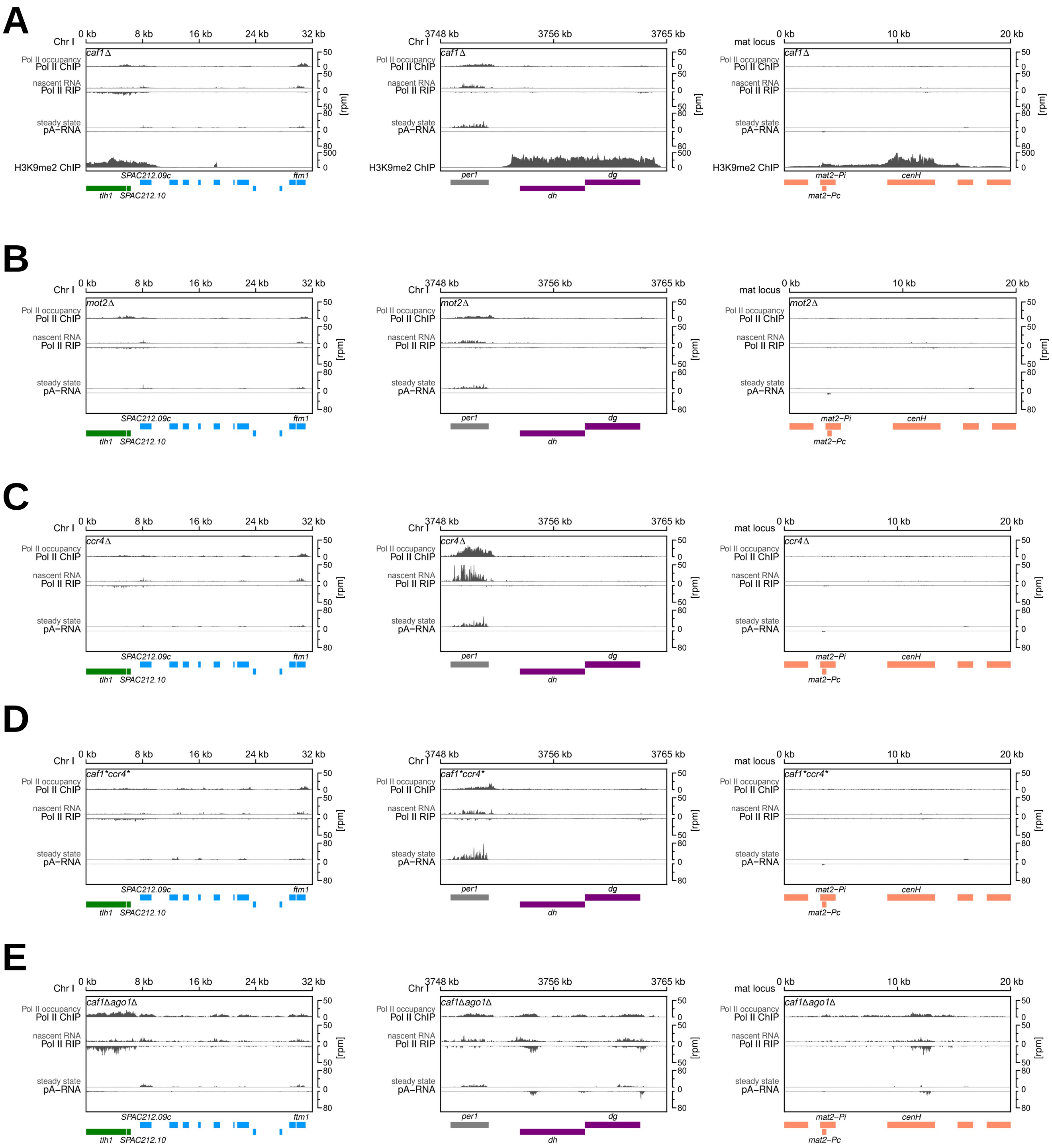

Supplemental Figure S6

Supplemental Figure S6: Next-Generation-Sequencing analysis

Next-Generation-Sequencing analysis of S2P-Pol II ChIP-seq (Pol II occupancy), S2P-Pol II RIP-seq (nascent RNA) and pA RNA-seq (steady state RNA) at subtelomeric and centromeric repeats, and at the *mat* locus. Locations of genes are indicated as boxes below the coverage according the color code: gray = protein coding; purple = *dg, dh*; green = *tlh, SPAC212.10*, blue = other heterochromatic genes; orange = *mat* locus.

(A) *caf1Δ* **(B)** *mot2Δ* **(C)** *ccr4Δ* **(D)** *caf1*ccr4** **(E)** *caf1Δago1Δ*

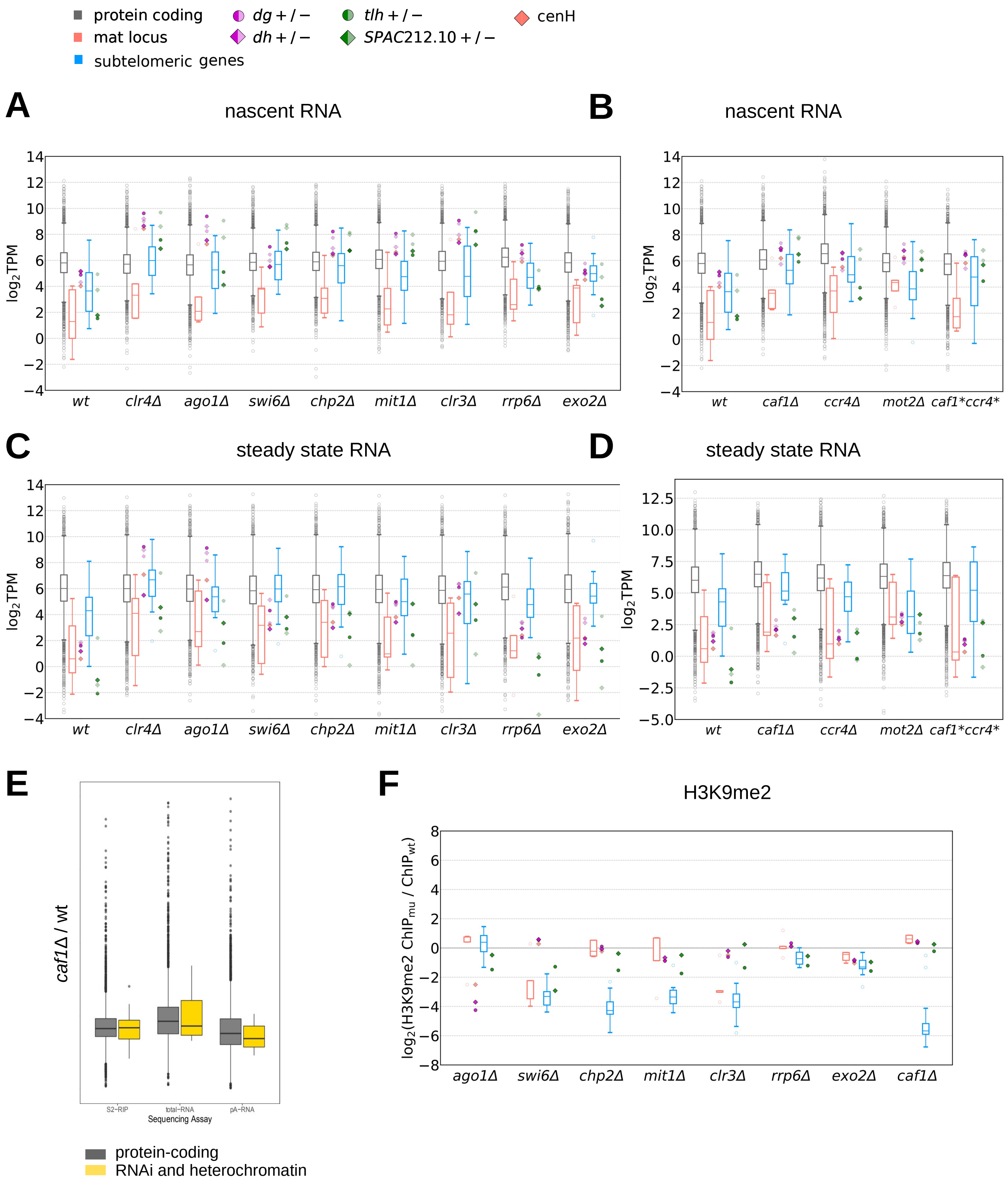

Supplemental Figure S7

Supplemental Figure S7:

(**A, B**) Box plot showing S2P-Pol II RIP-seq data in wild-type and mutant cells. Nascent RNA analysis is shown for individual mutants affecting (**A**) heterochromatin formation and RNA degradation or (**B**) the CCr4-Not complex. Data are plotted as defined for Figure 1B.

(**C, D**) pA RNA-seq results (steady state RNA) shown as box plot for individual wild-type and mutants affecting (**C**) heterochromatin formation and RNA degradation or (**D**) the CCr4-Not complex. Data are plotted as defined for Figure 1B.

(**E**) Box plot showing ratio of RNA levels in S2P-Pol II RIP-seq, total RNA and pA RNA data. Protein coding genes are shown in grey and genes involved in RNAi and heterochromatin formation are shown in yellow.

(**F**) Box plot showing H3K9me2 ChIP-seq data. H3K9me2 analysis is shown for individual mutants affecting heterochromatin formation or RNA degradation. Data are plotted as defined for Figure 1B. Average of at least two independent samples is shown.

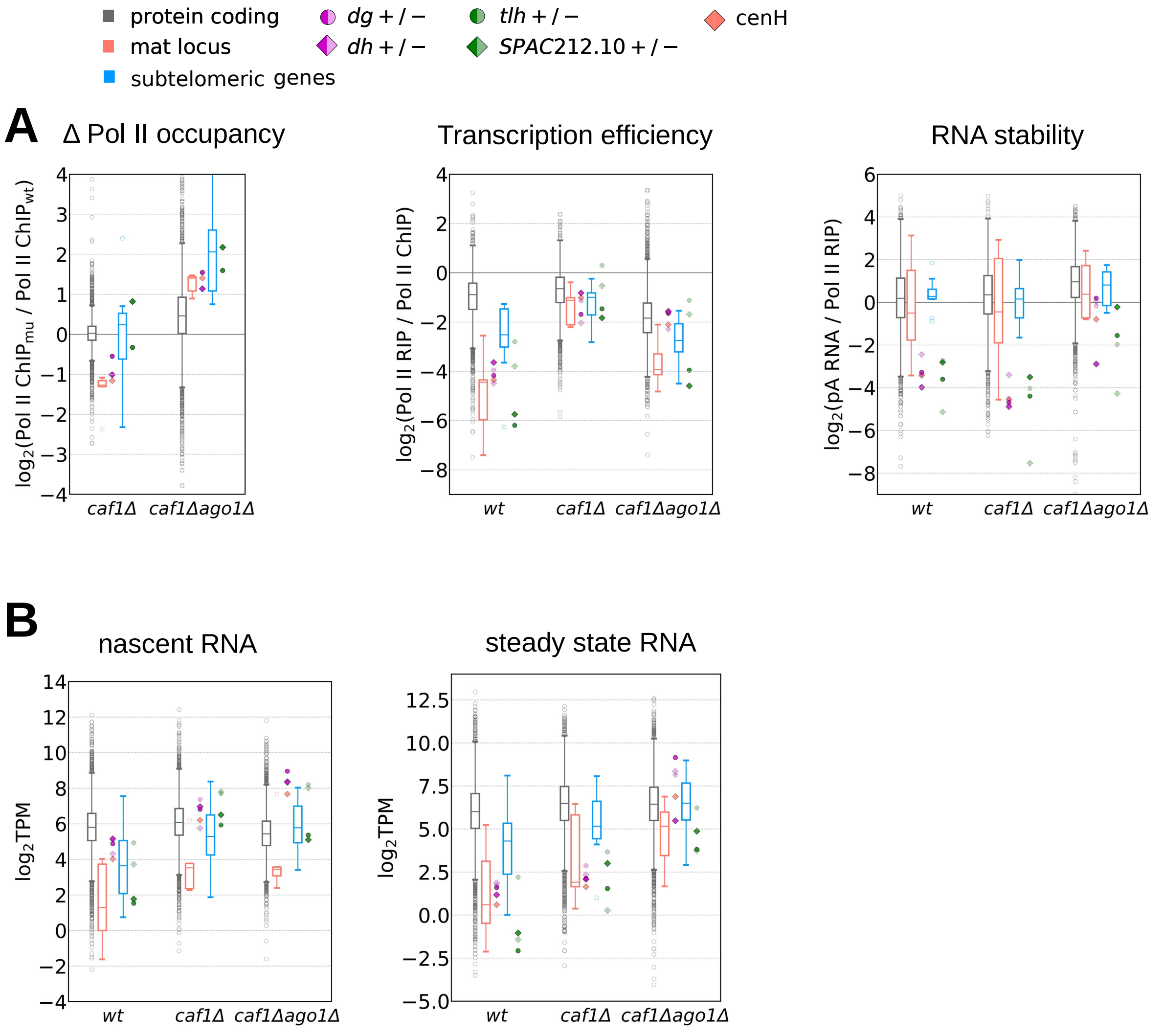

Supplemental Figure S8

Supplemental Figure S8:

(**A**) Box plots showing RNA Pol II occupancy, transcription efficiency and RNA stability over indicated genes in *caf1Δago1Δ* cells. Data are plotted as defined for Figure 1B.

(**B**) Box plots showing nascent and steady state RNA over indicated genes in *caf1Δago1Δ* cells. Data are plotted as defined for Figure 1B.

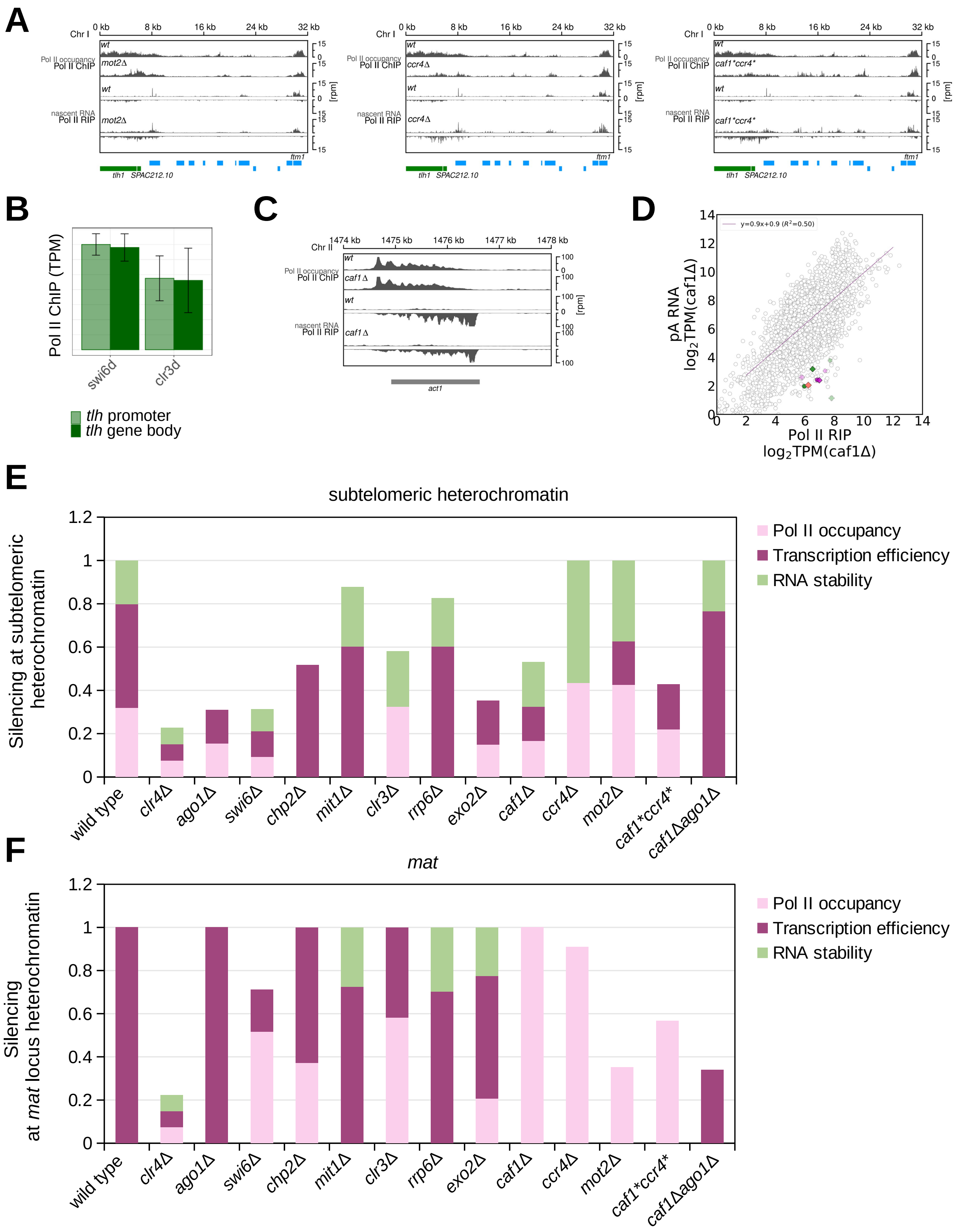

Supplemental Figure S9

Supplemental Figure S9:

(A) Analysis of the next-generation sequencing data showing occupancy of S2P RNA Pol II (ChIP-seq) and nascent RNA (S2P-Pol II RIP-seq) at subtelomeric regions in *S. pombe mot2Δ, ccr4Δ and caf1*ccr4** cells. Gene locations are indicated as boxes below the coverage and color-coded: green, subtelomeric loci *tlh* and *SPAC212.10*; blue, other subtelomeric genes.

(**B**) Quantification of RNA Pol II occupancy (S2P-Pol II ChIP-seq) at *tlh* promoter region and *tlh* gene body in indicated wild type and mutant strains.

(**C**) Analysis of the next-generation sequencing data showing occupancy of S2P RNA Pol II (ChIP-seq) and nascent RNA (S2P-Pol II RIP-seq) at euchromatic gene actin in *S. pombe caf1Δ* cells. Gene locations are indicated as boxes below the coverage.

(**D**) RNA stability in *caf1Δ* cells. pA RNA-seq (steady state RNA) data plotted over S2P-Pol II RIP-seq data (nascent RNA). TPM, transcripts per million. Gray circles are individual protein-coding genes; regression line is also shown in purple. Also plotted are centromeric *dg* and *dh* (dark purple for + strand, bright purple for - strand) and *tlh* and *SPAC212.10* (dark green for + strand, bright green for – strand) and cenH (orange). Each data point is the average of at least two independent samples.

(**E**) Bar chart displaying contribution of each pathway that is still active in the mutants to the silencing of other subtelomeric genes. The height of each bar corresponds to the fold change in RNA output relative to wild-type. The relative contribution of each pathway was computed as fold change in quantitative measures (ratios of average TPM, see Methods) relative to *clr4Δ*.

(**F**) Bar chart displaying contribution of each pathway that is still active in the mutants to the silencing at the *mat* locus silencing. The height of each bar corresponds to the fold change in RNA output relative to wild-type. The relative contribution of each pathway was computed as fold change in quantitative measures (ratios of average TPM, see Methods) relative to *clr4Δ*.

Supplemental Table S1: Strains used in this study

65	h90 otr1R(SphI)::ura4+ ura4-DS/E leu1-32 ade6-M210 natMX6::3xFLAG-*ago1*
63	h+ otr1R(SphI)::ura4+ ura4-DS/E leu1-32 ade6-M210
80	h+ otr1R(SphI)::ura4+ ura4-DS/E leu1-32 ade6-M210 *clr4Δ*::kanMX6
638	h+ otr1R(SphI)::ura4+ ura4-DS/E leu1-32 ade6-M210 *ago1Δ*::kanMX6
301	h90 mat3::ura4+ ura4-DS/E leu1-32 ade6-M210 *swi6Δ*::natMX6
324	h90 mat3::ura4+ ura4-DS/E leu1-32 ade6-M210 *chp2Δ*::kanMX6
491	h+ leu1-32 ura4-D18 imr1R(NCol)::ura4+ oriI ade6-216 *mit1Δ*::kanMX6
302	h+ otr1R(SphI)::ura4+ ura4-DS/E leu1-32 ade6-M210 *clr3Δ*::TAP-kanMX6
504	h+ otr1R::ura4, ura4-DS/E, ade6-M216; leu1-32, his7-366 natMX6::3xFLAG-*ago1* *rrp6Δ*::kanMX6
530	h+ otr1R(SphI)::ura4+ ura4-DS/E leu1-32 ade6-M210 natMX6::3xFLAG-*ago1* *exo2Δ*::kanMX6
510	h90 otr1R(SphI)::ura4+ ura4-DS/E leu1-32 ade6-M210 natMX6::3xFLAG-*ago1* *caf1*Δ::kanMX6
591	h90, ade6-D1, his3-D1, leu1-3, ura4-D18, otr1R(SphI)::ade6$^+$, TAS-his3$^+$-tel1(L), TAS-ura4$^+$-tel2(L), *caf1*Δ::kanMX6
544	h90 otr1R(SphI)::ura4+ ura4-DS/E leu1-32 ade6-M210 natMX6::3xFLAG-*ago1* *ccr4*Δ::hphMX6
1168	h90, ade6-D1, his3-D1, leu1-3, ura4-D18, otr1R(SphI)::ade6$^+$, TAS-his3$^+$-tel1(L), TAS-ura4$^+$-tel2(L), ccr4H664A-ccr4Terminator::hphMX6, nat::caf1promoter-caf1D53AD243AD174A
1022	h90 otr1R(SphI)::ura4+ ura4-DS/E leu1-32 ade6-M210 natMX6::3xFLAG-*ago1* *mot2*Δ::kanMX6

1023	h90 otr1R(SphI)::ura4+ ura4-DS/E leu1-32 ade6-M210 natMX6::3xFLAG-*ago1* *mot2*Δ::kanMX6
523	h90 otr1R(SphI)::ura4+ ura4-DS/E leu1-32 ade6-M210 *caf1*Δ::kanMX6 *ago1*Δ::hph

Supplemental Table S2: List of heterochromatic genes

subtelomeric genes : 'SPAC212.09c', 'SPNCRNA.70', 'SPAC212.08c', 'SPAC212.07c', 'SPAC212.12', 'SPAC212.06c', 'SPAC212.04c', 'SPAC212.03', 'SPAC212.02', 'SPAC212.01c', 'SPAC977.01', 'SPAC977.18', 'SPAC977.02', 'SPAC977.03', 'SPAC977.04', 'SPAC212.05c'

***mat* locus :** 'SPMTR.01', 'FP565355_region_1..2120', 'FP565355_region_9170..13408', 'FP565355_region_15609..16735', 'FP565355_region_18009..20128'
